# Supplementary figures and images for: Concomitant Chemoradiation Therapy with Gold Nanoparticles and Platinum Drugs Co-Encapsulated in Liposomes
Source: Int J Mol Sci. 2020 Jul 9;21(14):4848. doi: 10.3390/ijms21144848 (PMC7402338; doi:10.3390/ijms21144848)

[illegible][illegible]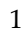

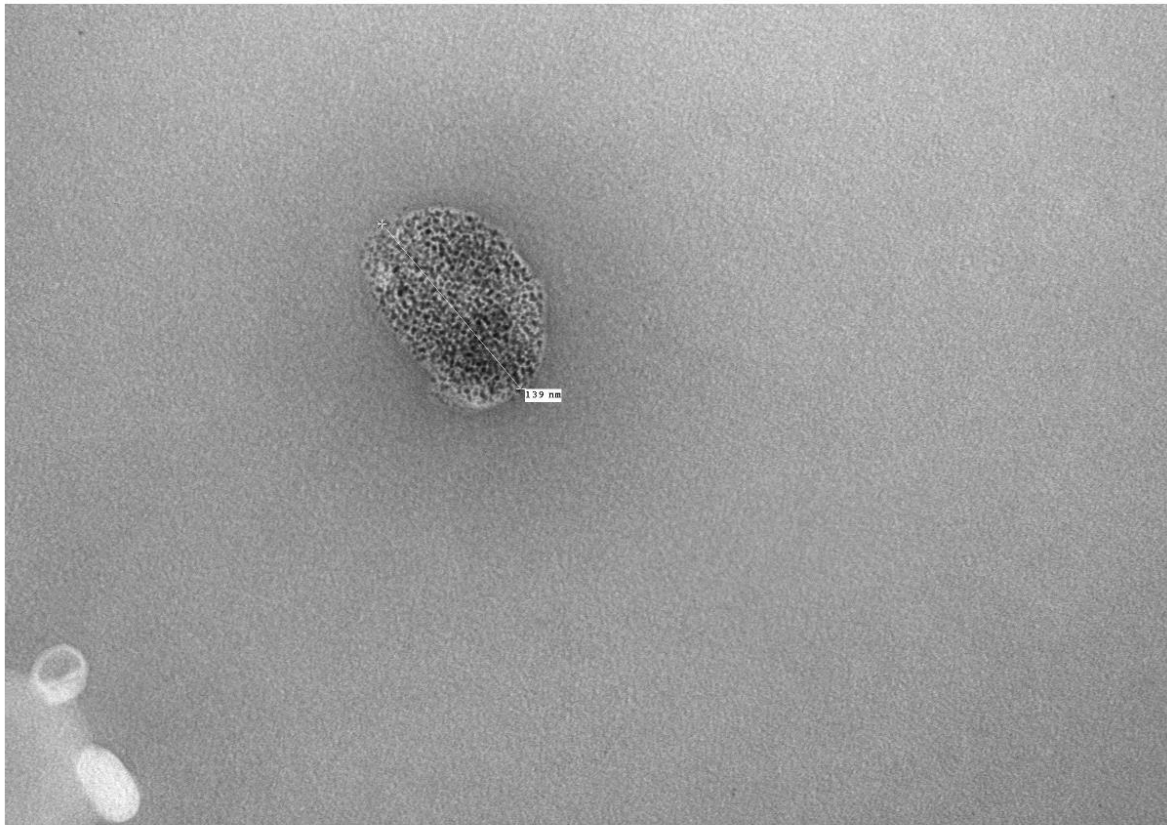

LipoGold TEM image

20 nm  
HV=60.0kV  
Direct Mag: 80000x  
Universite de Sherbrooke

Supplement: Supplementary file 1 [file ijms-21-04848-s001.pdf]
